# Is There a Neuropathic-Like Component to Endometriosis-Associated Pain? Results From a Large Cohort Questionnaire Study

**DOI:** 10.3389/fpain.2021.743812

**Published:** 2021-11-04

**Authors:** Lydia Coxon, Katja Wiech, Katy Vincent

**Affiliations:** ^1^Nuffield Department of Women's and Reproductive Health, University of Oxford, Oxford, United Kingdom; ^2^Nuffield Department of Clinical Neurosciences, Wellcome Centre for Integrative Neuroimaging, University of Oxford, Oxford, United Kingdom

**Keywords:** endometriosis, neuropathic pain, painDETECT, endometriosis-associated pain, depression, anxiety, surgery, questionnaire

## Abstract

**Background:** Pain is one of the primary symptoms of endometriosis, a chronic inflammatory condition characterised by the presence of endometrial tissue outside the uterus. Endometriosis-associated pain is commonly considered as nociceptive in nature, but its clinical presentation suggests that it might have neuropathic-like properties in a subgroup of patients.

**Methods:** This is a cross sectional study using an online survey. The survey was distributed by patient support websites. The survey was composed of validated questionnaires assessing pain symptoms, psychological measures and questions about number of surgeries.

**Main Results:** We had 1,417 responses which met the inclusion criteria. Using standard painDETECT cut-off scores, we found that pain was classified as neuropathic in 40% of patients and as mixed neuropathic/nociceptive in a further 35%. In line with observations in other neuropathic conditions, the neuropathic subgroup reported higher pain intensities, greater psychological distress and cognitive impairment. Neuropathic pain was also more likely in those with more surgeries to the abdomen and a longer history of pain. As revealed by a cluster analysis, those with a neuropathic pain component could further be divided into two subgroups based on their sensory profile.

**Conclusions:** The data presented here indicate that endometriosis-associated pain includes a neuropathic-like component in a substantial proportion of women. Although further investigation is required, our finding challenges the current conceptualisation of endometriosis-associated pain as nociceptive and advocates for a new perspective on this type of pain, which is so debilitating to a large number of women.

## Introduction

Endometriosis is a chronic inflammatory condition, characterised by the presence of endometrial tissue outside of the uterus (1). Pain is a primary symptom, with dysmenorrhea, dyspareunia and non-cyclical pelvic pain being the most prevalent pain symptoms (2). There is poor correlation between pain severity and disease burden and knowledge regarding biological mechanisms giving rise to pain is still sparse (3). Currently available treatments focus on surgical excision/ablation of the lesions or hormonal suppression. Both of these are associated with risks and side effects, and have been linked to persistent or recurrent pain in a large proportion of women (4).

Clinically, women often describe their endometriosis-related pain as “stabbing” and “tingling,” which are known to be key features of neuropathic pain; however, the prevalence of a neuropathic-like component has not been properly investigated in this population. Neuropathic pain is defined by the International Association for the Study of Pain as “pain caused by disease or lesion of the somatosensory nervous system,” contrasting with nociceptive pain which is defined as “pain that arises from actual or threatened damage to non-neural tissue and is due to the activation of nociceptors” (5). Neuropathic pain could be expected to arise in the context of endometriosis for a number of reasons. Firstly, there is currently no non-invasive test to establish the diagnosis of endometriosis. Consequently, all women with a confirmed diagnosis will have undergone at least one surgical procedure, with the associated risk of post-surgical neuropathic pain (6–8). Secondly, endometriotic lesions are themselves innervated (2, 9). Surgical procedures excising/ablating the lesions may damage these nerve fibres, which could generate neuropathic pain (7). Thirdly, these nerve fibres express TRPV1 receptors and are bathed in peritoneal fluid, known to contain high levels of inflammatory mediators such as BDNF and TNF-alpha in women with endometriosis, potentially sensitising nerve endings (2, 10). Thus, neuropathic pain could develop as a result of prolonged exposure to these inflammatory mediators (10). In a recent study on chronic pelvic pain which included a subset of patients with endometriosis (*n* = 32) (11), over 50% of patients showed clinical features of neuropathic pain. There is ample evidence from both animal and human studies showing that neuropathic and nociceptive pain differ considerably, including with respect to their underlying pathology (12, 13), cognitive-affective processing (14, 15), and responses to treatment (12). Therefore, a better understanding of the prevalence of neuropathic pain in endometriosis could guide both clinical care and future clinically oriented research strategies.

Here, we investigated the prevalence of neuropathic-like pain in endometriosis using a questionnaire screening tool, painDETECT. We hypothesised that endometriosis-associated pain would be classified as neuropathic in a subgroup of women. As in other types of neuropathic pain (16), we hypothesised that these women would have higher depression and anxiety scores. We also expected a higher proportion of women with neuropathic-like pain the more surgeries they had had to the abdomen. Studies in other neuropathic pain conditions have used cluster analysis to identify subtypes of neuropathic pain which differ in their sensory profile (using painDETECT responses) (17, 18). We used a similar strategy to test for subgroups amongst those within our cohort classified as having neuropathic or mixed pain.

## Methods

### Survey Design

To target a large sample from across the UK with endometriosis associated pain, we designed an online survey using painDETECT as a screening tool for neuropathic-like pain and hosted this on patient support websites (for full survey see Supplementary Material).

The survey was created using Online Surveys (https://www.onlinesurveys.ac.uk) and was designed to be self-completed without professional guidance by combining single questions with validated questionnaires. Questions included patients' ratings of the maximum pain intensity experienced over the last 12 months, for dysmenorrhea, dyspareunia (no time frame specified), non-cyclical pain, dyschezia and dysuria using a Numerical Rating Scale (NRS) anchored at 0 = no pain and 10 = worst imaginable pain; duration of each type of pain since onset of symptoms (years) and number of surgeries received to the abdomen.

For somatosensory symptoms of neuropathic pain, painDETECT (19, 20) was used which was designed and validated as a screening tool for neuropathic pain. This validated questionnaire consists of nine questions, which assess the severity, course, quality and nature of the patient's pain. For example, the questions require the patient to rate the intensity of symptoms including spontaneous burning pain and pain evoked by light pressure, the pain course pattern, and to indicate whether their pain radiates to other parts of the body. Intensity ratings for symptoms are scored between 0 (never), 1 (hardly noticed), 2 (slightly), 3 (moderately), 4 (strongly) or 5 (very strongly). The total painDETECT score ranges between −1 and 38. PainDETECT standard cut-off scores were used to group participants into those with nociceptive (≤12), mixed (13–18) and neuropathic-like (≥19) pain (19). This categorisation has been shown to correspond to clinical diagnoses of neuropathic pain (e.g., neurological examination) in various chronic pain populations, including those not traditionally considered neuropathic (21).

To assess a “centralised” pain component, we included the Fibromyalgia Symptom Scale (FS) (22) (scores are between 0 and 31) which assesses whether pain is widespread or localised and whether patients experience related symptoms. Although originally designed for fibromyalgia patients, the FS questionnaire has additionally been used in patients with postoperative pain following hysterectomy (23) as well as in other pain conditions (24–26).

For a specific assessment of symptoms of depression and anxiety we included the Beck Depression Inventory (BDI) (total score: 0–63) (27); and State Trait Anxiety Inventory, Trait (STAI-T) (total score: 20–80) (28).

Finally, the Pain Sensitivity Questionnaire (PSQ) was included as a patient-rated assessment of their pain sensitivity to hypothetical stimuli (0 = not painful at all to 10 = worst pain imaginable) (29). PSQ ratings have been shown to be positively correlated with experimental pain intensity ratings and pain thresholds in healthy volunteers and chronic pain patients (29, 30).

All measures described were only scored if all questions for that measure were answered [e.g., if participant did not answer “Do you suffer from a burning sensation (e.g., stinging nettles) in the are(a) of your pain?” they were not given a painDETECT score or classified as nociceptive/mixed/neuropathic].

The survey was posted on patient support websites (Endometriosis UK, www.endometriosis-uk.org; Endometriosis Association of Ireland, www.endometriosis.ie; and Endometriosis SHE Trust UK, www.facebook.com/EndoSheTrust/). It was open for responses between March and May 2018. Participants could complete the survey at their leisure, in several sessions and were able to withdraw at any time. The survey was beta tested on 9 patients, recruited from clinics at the Oxford University Hospitals Foundation Trust and the survey was modified according to their feedback. Patients were not reimbursed for their participation.

### Ethical Approval

This study was approved by the Central University Research Ethics Committee, University of Oxford, R56567/RE002. Implied consent was attained via tick boxes to ensure the participant was over the age of 18 and that they agreed to take part in study.

### Data Analysis

All statistical analysis was carried out using SPSS Statistics version 25. Participants were excluded if they had not received laparoscopic surgery (needed to diagnose endometriosis) or if they had not provided the age at which they received the diagnosis. The duration of pain symptoms was calculated as the difference between participants' current age and the time-point they first experienced the specific symptom (e.g., 15 years old for dysmenorrhea). Maximum duration of pain was defined as the longest duration of pain symptoms for either dysmenorrhea, dyspareunia or non-cyclical pain.

To explore whether neuropathic pain is related to higher pain intensity, we compared pain intensity ratings between the three painDETECT groups separately for each pain type. BDI scores and STAI-T scores were compared between the neuropathic, mixed and nociceptive groups. To explore the relationship between painDETECT and (i) FS Score and (ii) PSQ Scores, correlation coefficients were calculated.

The number of surgeries to the abdomen was used as a grouping variable: group 1 had one surgery, group 2 had two surgeries, group 3 had three surgeries, group 4 had four surgeries and group 5 had five or more surgeries. To test whether neuropathic-like pain was more prevalent in those with more surgeries, we compared the proportion of patients classified as having neuropathic pain between groups 1 and 5.

As most variables were not normally distributed (indicated by significant Kolmogorov-Smirnov tests), we chose to use non-parametric tests throughout. Group differences were explored using Kruskal-Wallis tests; significant results were followed up with Mann-Whitney U tests. Multiple comparison correction was carried out (Bonferroni correction). Correlation analyses were performed using Spearman's correlations. Additionally, we calculated partial correlations controlling for pain intensity. Rho values are interpreted such that rho <0.3 is “weak”; rho between 0.4 and 0.69 are “moderate”; rho between >0.7 are “strong” (31).

#### Cluster Analysis

Sensory symptom profiles were determined based on seven cardinal symptoms assessed in the painDETECT questionnaire as previously described (17, 18). These include

- Do you suffer from a burning sensation (e.g., stinging nettles) in the area(s) of your pain?
- Do you have a tingling or prickling sensation in the area of your pain (like crawling ants or electrical tingling)?
- Is light touching (clothing, a blanket) in this are painful? Do you have sudden pain attacks in the area of your pain, like electric shocks?
- Is cold or heat (bath water) in this area occasionally painful?
- Do you suffer from a sensation of numbness in this area?
- Does slight pressure in this area, e.g., with a finger, trigger pain?

Responses of participants categorised as having mixed and neuropathic pain were entered into a two-step cluster analysis. To account for inter-individual differences in pain sensitivity, painDETECT scores for each of these symptoms were recalculated by subtracting the mean across all seven responses from each individual response as described in Baron et al. (17). Scores larger than zero thereby indicate a sensation that is more intense than the average individual symptom score.

Two-step cluster analysis with log-likelihood as distance measure was used with these continuous variables. Note that previous studies had employed a slightly different approach in their cluster analysis (17, 18). We used a two-step cluster analysis with the optimal number of clusters determined using the Schwarz Bayesian Criterion. In addition to the number of clusters identified, we report the quality of the suggested solution based on the silhouette coefficient which jointly considers cluster cohesion (i.e., how close items are within one cluster) and separation (i.e., how well each cluster is separated from the others). The silhouette coefficient can range from −1 (indicating that samples have been assigned to the wrong cluster) to 1 (indicating that the sample is far from the neighbouring cluster). Categorisation of the silhouette coefficient into poor, fair or good is based on work by Kaufman et al. (32). Furthermore, we report the importance of each symptom for cluster formation (predictive importance).

Non-parametric Mann-Whitney U tests were used to assess differences between clusters with respect to pain and psychological measures.

## Results

One thousand six hundred fifteen responses were received, with 1,417 meeting the inclusion criteria. Participant demographics can be seen in Table 1.

**Table 1:** Demographics of participants.

	**Median (range) (IQR)**
Age (years)	33 (18–59) (27–38)
Dysmenorrhea numerical rating scale score (0–10) (*n* = 1,397, 98.6%) (2.7% reported a rating of 0)	8 (0–10) (7–9)
Dyspareunia numerical rating scale score (0–10) (*n* = 1,404, 99.1%) (10.2% reported a rating of 0)	7 (0–10) (5–8)
Non-cyclical pain numerical rating scale score (0–10) (*n* = 1,323, 93.4%) (6.3% reported a rating of 0)	8 (0–10) (6–9)
Maximum duration of pain symptoms (years)	18 (1–47) (13–24)
Dysuria	4 (0–10) (0–7)
Dyschezia	7 (0–10) (5–9)
Number of surgeries to the abdomen	2 (1–23) (1–4)

*Median values, range and interquartile range (IQR) of responses given as data not normally distributed. Number of participants (and as a percentage of total participants) giving a NRS score for each type of pain is given. Two percentage gave NRS scores of 0 for two of the pain types, meaning they experienced only one type of pain (e.g., dyspareunia only), 14.7% gave NRS scores > 0 for two types of pain (e.g., they experienced dysmenorrhea and non-cyclical pain), 83.3% gave NRS scores > 0 for all three main pelvic pain symptoms (dysmenorrhea, dyspareunia, and non-cyclical pain). Maximum duration of pain symptoms is defined as the longest duration of either dysmenorrhea, dyspareunia or non-cyclical pain*.

Of the 1,417 participants, *n* = 13 were missing for painDETECT score, *n* = 29 were missing FS, *n* = 28 were missing PSQ, *n* = 46 were missing BDI and *n* = 87 were missing STAI-T scores.

Of the resulting *n* = 1,401, 40% (*n* = 558) were categorised as having neuropathic pain according to their painDETECT scores. Pain was classified as mixed nociceptive/neuropathic in a further 35% (*n* = 488) and as nociceptive in the remaining 25% (*n* = 358).

To test whether the classification of pain as neuropathic was related to more intense pain, we compared intensity ratings for all relevant types of pain between the three groups. These analyses showed significant differences in NRS scores for dysmenorrhea [χ^2^(2) = 91.710, *p* = 6.088 × 10^−20^], dyspareunia [χ^2^(2) = 80.191, *p* < 0.001], non-cyclical pain [χ^2^(2) = 96.884, *p* < 0.001], dyschezia [χ^2^(2) = 70.162, *p* < 0.001] and dysuria [χ^2^(2) = 117.853, *p* < 0.001]. For all pain types, scores were highest for the neuropathic group followed by the mixed group. *Post-hoc* tests showed that all pairwise group comparisons reached statistical significance (*p* < 0.001) with the exception of non-cyclical pain for which the difference between nociceptive and mixed groups did not withstand multiple comparison correction (*p* = 0.027 uncorrected).

Correlation analysis between painDETECT scores and FS and PSQ, showed a moderate correlation between painDETECT and FS scores (rho = 0.441, *p* < 0.001) and no significant correlation between painDETECT and PSQ.

Scores for depression and anxiety were significantly different between groups [χ^2^(2) = 130.907, *p* < 0.001; χ^2^(2) = 67.389, *p* < 0.001, respectively] (see Table 2). Fatigue and trouble thinking/remembering were significantly different between groups [χ^2^(2) = 85.219, *p* < 0.001, χ^2^(2) = 68.349, *p* < 0.001, respectively]. Additionally the neuropathic group was more likely to report waking up feeling tired [χ^2^(2) = 80.588, *p* < 0.001]. *Post-hoc* tests showed significant differences in all pairwise comparisons between groups, with the neuropathic group showing the strongest impairment followed by the mixed group (*p* < 0.05 for all *post-hoc* comparisons) (see Table 2).

**Table 2:** Age, numerical rating scale scores, scores of cognitive and affective measures for participants in each painDETECT group (neuropathic, mixed, and nociceptive).

	**Neuropathic (median, IQR)** **(*n* = 558)**	**Mixed (median, IQR)** **(*n* = 488)**	**Nociceptive (median, IQR)** **(*n* = 358)**	****χ^2^(2)**, *p***
Age (years)	32 (27–38)	33 (27–37)	34 (29–40)	16.539, <0.001
Maximum duration of pain (years)	18 (12.5–24)	18 (12–23.75)	18.5 (13–24)	2.244, 0.326
Dysmenorrhea	9 (8–10)	8 (7–9)	8 (7–9)	91.710, <0.001
Duration of dysmenorrhea (years)	18 (13–24)	18 (12–23)	18 (14–24.75)	3.208, 0.201
Dyspareunia	7 (6–9)	7 (5–8)	6 (4–8)	80.191, <0.001
Duration of dyspareunia (years)	10 (5–15)	9 (5–14)	9 (5–14)	3.985, 0.136
Non-cyclical pain	8 (7–10)	8 (6–9)	7 (5–8)	96.884, <0.001
Duration of non-cyclical pain (years)	11 (6–17)	9 (5–17)	9 (5–15)	16.806, <0.001
Dyschezia	8 (6–9)	7 (5–9)	6 (4–8)	70.162, <0.001
Dysuria	6 (2–7)	4 (0–6)	0 (0–5)	117.853, <0.001
Depression (BDI)	28 (19–38)	24 (16–34)	16 (11–26)	130.907, <0.001
Anxiety (STAI-T)	55 (47–64)	53 (45–61)	48 (38–56)	67.389, *p* < 0.001
Fatigue	3 (2–3)	2 (2–3)	2 (2–3)	85.219, <0.001
Trouble thinking/remembering	2 (1–3)	2 (1–2)	2 (1–2)	68.349, <0.001
Waking up feeling tired	3 (2–3)	3 (2–3)	2 (2–3)	80.588, <0.001

*Maximum duration of pain is defined as the longest duration of either dysmenorrhea, dyspareunia or non-cyclical pain. IQR, Interquartile range. χ^2^, p from Kruskal-Wallis test. Post-hoc tests of significant results showed that all pairwise group comparisons reached statistical significance (p < 0.001) with the exceptions of: non-cyclical pain for which the difference between nociceptive and mixed groups did not withstand multiple comparison correction (p = 0.027 uncorrected); and age for which the comparison between the mixed and neuropathic groups was not significant (p = 0.807)*.

Correlation analyses revealed a significant positive correlation between painDETECT scores and each of the cognitive-affective variables, even when controlling for pain scores (Table 3).

**Table 3:** Correlations between painDETECT score and cognitive-affective variables.

	**BDI**	**STAI**	**Fatigue**	**Trouble thinking/remembering**	**Waking up tired**
painDETECT (standard correlations)	0.33^*^	0.24^*^	0.26^*^	0.24^*^	0.26^*^
painDETECT (partial correlations)	0.27^*^	0.19^*^	0.19^*^	0.18^*^	0.19^*^

*Standard correlations show Spearman's correlation coefficients. Partial correlations control for Numerical Rating Scale scores for dysmenorrhea, dyspareunia and non-cyclical pain*.

* *p < 0.001;*

*BDI, Beck Depression Inventory; STAI-T, State-Trait Anxiety Inventory*.

**Figure 1:**
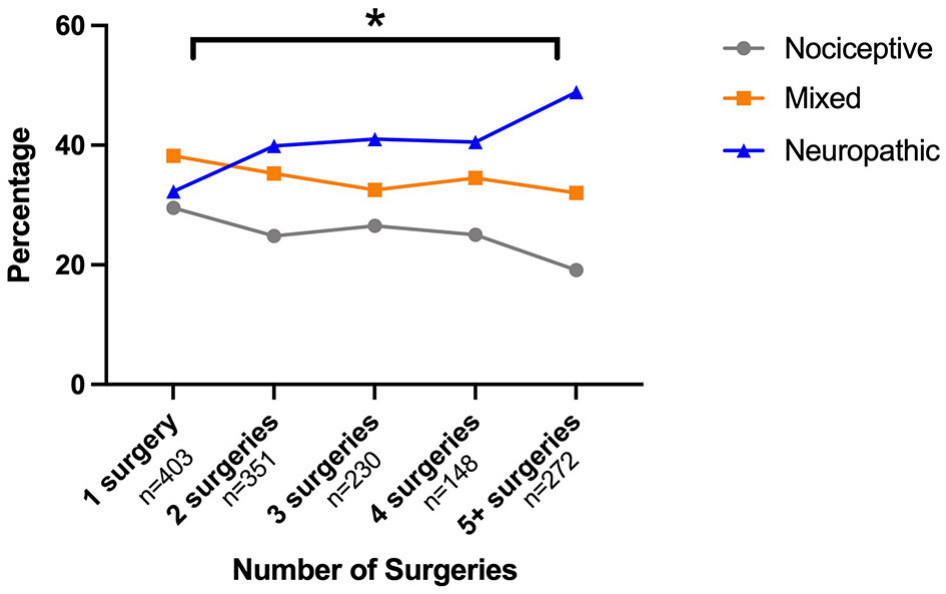
Relationship between painDETECT scores and number of abdominal surgeries. Surgeries were grouped so that each group had approximately the same number of participants. Surgery group 1 had one surgery to the abdomen, group 2 had two surgeries, group 3 had three to four surgeries and group 4 had five or more surgeries. *Post-hoc* tests showed significant differences between group 1 and group 4 in painDETECT scores (*p* < 0.001).

Comparing painDETECT scores between groups with increasing numbers of surgical procedures revealed significant group differences [χ^2^(4) = 18.963, *p* < 0.001] (see Figure 1). *Post-hoc* tests confirmed that group 5 with five and more surgeries had a significantly higher painDETECT score than group 1 that had undergone one surgery (*p* < 0.001).

Exploring the relationship between duration of pain and painDETECT group, significant group differences were only found for duration of non-cyclical pain [χ^2^(2) = 16.806, *p* < 0.001]. *Post-hoc* tests showed that patients in the neuropathic group had experienced non-cyclical pain for longer than those in the mixed (*p* = 0.006) and nociceptive (*p* < 0.001) groups.

### Sensory Symptom Profiles and Cluster Analysis

To explore the sensory symptom profile of those categorised as having neuropathic or mixed pain, sensory symptoms rated in painDETECT were analysed in more detail. Table 4 shows the proportion of participants that had clinically relevant sensory disturbances (scores > 3; strongly, very strongly). The presence of painful attacks was by far the most common symptom seen in this cohort.

**Table 4:** Reported symptoms in neuropathic and mixed groups.

**Sensory symptom**	**Proportion of patients affected**
Burning	28.2%
Prickling	25.4%
Mechanical allodynia	23.3%
Painful attacks	79.3%
Thermal hyperalgesia	8.9%
Numbness	14.1%
Pressure evoked pain	45.2%

*Proportion of participants in the neuropathic and mixed groups reporting clinically significant symptoms (i.e., a score of >3, strongly or very strongly) in the painDETECT questionnaire*.

**Figure 2:**
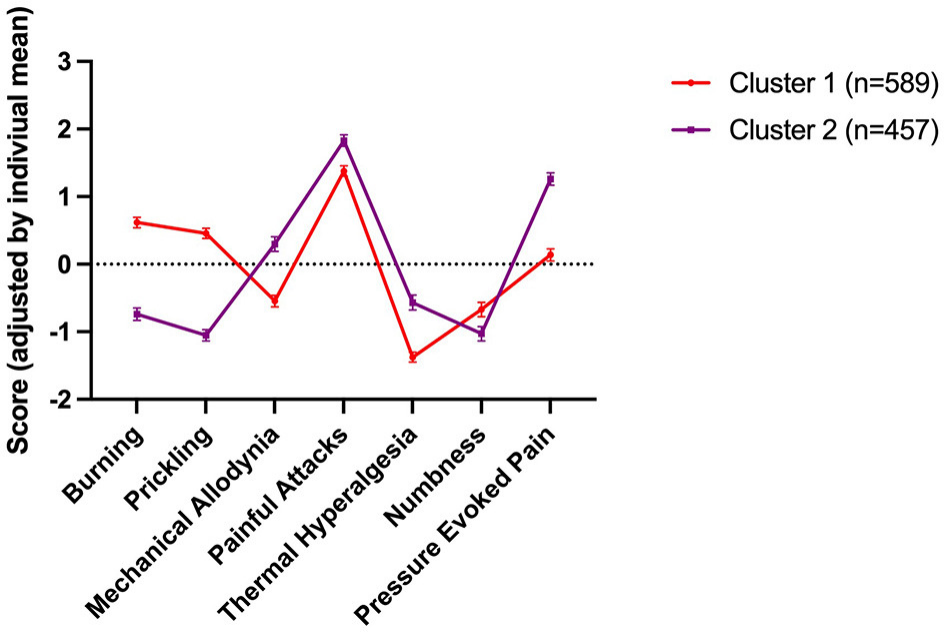
Sensory symptom profile of the two clusters. Scores were individually mean adjusted. The cluster model had a silhouette measure of cohesion and separation of 0.3, indicating it was of a “fair” quality. 56.3% of patients fell into cluster 1 (*n* = 589), and 43.7% in cluster 2 (*n* = 457). The symptoms with the greatest predictive importance were “prickling/tingling” (predictive importance = 1), and “burning” (predictive importance = 0.77), which were most characteristic for cluster 1. In contrast, “pressure evoked pain” (predictive importance = 0.48), “thermal hyperalgesia” (predictive importance = 0.27) and “mechanical allodynia” (predictive importance = 0.27) were more common in cluster 2. “Painful attacks” (predictive importance = 0.11) and “numbness” (predictive importance = 0.05) were least discriminatory.

To determine if those categorised as having neuropathic or mixed pain could be divided into subgroups, we performed a cluster analysis based on their sensory symptom profiles. The analysis produced two clusters (Figure 2). Scores were individually mean adjusted. The cluster model had a silhouette measure of cohesion and separation of 0.3, indicating it was of a “fair” quality. 56.3% of patients fell into cluster 1 (*n* = 589), and 43.7% in cluster 2 (*n* = 457). The symptoms with the greatest predictive importance were “prickling/tingling” (predictive importance = 1), and “burning” (predictive importance = 0.77), which were most characteristic for cluster 1. In contrast, “pressure evoked pain” (predictive importance = 0.48), “thermal hyperalgesia” (predictive importance = 0.27) and “mechanical allodynia” (predictive importance = 0.27) were more common in cluster 2. “Painful attacks” (predictive importance = 0.11) and “numbness” (predictive importance = 0.05) were least discriminatory.

There were no significant differences between clusters with respect to depression (BDI), anxiety (STAI-T), fatigue, trouble thinking/remembering or waking up feeling tired (all *p* > 0.05).

Clusters did not differ significantly with respect to the intensity of dysmenorrhea, dyspareunia or non-cyclical pain and PSQ scores (*p* > 0.05) but FS scores were significantly higher in cluster 1 (*p* < 0.001).

## Discussion

Our data suggest that a considerable proportion of women with endometriosis-associated pain may have a neuropathic-like component to their pain. Based on painDETECT scores, pain was categorised as neuropathic in 40% of patients and as mixed (i.e., neuropathic and nociceptive pain) in a further 35% of the sample. In line with other chronic pain conditions where a mixture of underlying mechanisms, including neuropathic pain, is found (24, 33, 34) women who were classified as having neuropathic-like pain also report higher pain intensity scores, more psychological distress and alterations in cognitive processing (Table 2). Interestingly, we found that women who had undergone more surgical procedures had higher painDETECT scores, and those classified as having neuropathic pain had a longer duration of non-cyclical pain than those in the other groups, however our data is not suited to establish a causal relationship. Based on their sensory profile, those classified as having a neuropathic component could be further divided into two subgroups, suggesting that there may be more than one mechanism underlying neuropathic-like pain in women with endometriosis.

### Potential Mechanisms

As described, there are a number of potential mechanisms by which endometriosis may be associated with neuropathic-like pain. Here, we identified two factors related to the likelihood of neuropathic-like pain, namely the duration of non-cyclical pain and number of abdominal/pelvic surgeries (Figure 2). These relationships may simply represent failure of standard treatments when a neuropathic-like component is present, or they may in themselves be directly driving the neuropathic-like component. There is a growing body of evidence suggesting that surgery can generate neuropathic pain (6, 8, 35), with it being more likely in women, those with pre-operative pain, psychological distress or inflammation (36, 37); all of which are frequently present in those undergoing surgery for endometriosis.

Our subsequent analyses do suggest that the variation in underlying mechanisms may be more limited in neuropathic-like pain associated with endometriosis than in other neuropathic conditions. For example, the majority (79%) of our participants reported painful attacks (as opposed to 32% in painful radiculopathy and 46% in postherpetic neuralgia) (18). Furthermore, our cluster analysis (Figure 2) suggests that only two distinct subgroups of women exist.

### Clinical Relevance

Whilst endometriosis-associated pain syndrome is defined in the IASP taxonomy of pain (38), clinically endometriosis is still predominantly managed by gynaecologists and the pain symptoms considered to arise either from the ectopic tissue implants themselves or the inflammatory environment of the pelvis. Thus, current guidelines (4, 39, 40) all recommend simple analgesics (non-steroidal anti-inflammatories) and either hormonal suppression or surgical ablation/excision of the lesions. Pain management programmes are usually recommended only when treatment has failed. Many women with endometriosis-associated pain undergo repeated surgeries in the belief that pain symptoms may improve. A recent retrospective study found that over half the sample (*n* = 486) had undergone > 1 surgical procedure related to their endometriosis in a 10 year period (41). All these interventions are based on the understanding that endometriosis-associated pain is nociceptive in nature. However, our data indicate that, in line with other conditions associated with chronic pain [e.g., rheumatoid arthritis (34), lower back pain (21, 42) and bladder pain syndrome (33)], a neuropathic-like component exists for a large proportion of women. As neuropathic pain is now considered a specific multi-aetiology entity, with the efficacy of systemic drug treatments not depending on the underlying cause (13, 43), we believe that future work should be undertaken to determine whether such drugs can provide analgesia in women with endometriosis-associated pain.

However, follow-up studies with clinical examinations are needed to verify our findings and further explore the influence of additional variables (e.g., patient race, stage or location of the disease, endometriosis characteristics, characteristics of the menstrual cycle, use of contraceptives, comorbidities such as other pain conditions or uterine/pelvic pathologies) which were not assessed here.

Our findings have direct implications for the treatment of endometriosis-associated pain. The high prevalence of neuropathic-like pain in our large sample clearly argues for a consideration of this type of pain, at the very least in those women with recurrent/persistent symptoms. The painDETECT questionnaire is a short and easy to complete screening tool used and validated in several chronic pain conditions (21), which could be integrated into any standard gynaecology consultation. This is particularly true for those in whom a further surgical procedure is being considered. Whilst cross-sectional data such as this cannot determine underlying mechanisms or the direction of relationships, the strong relationship between the number of surgeries and a neuropathic-like component suggests that either surgery is not effective at treating this type of pain (leading to repeated procedures) or that it is in itself involved in generating this pain. Alternative strategies should therefore be considered first, unless there is another indication for surgery (e.g., pelvic mass). Furthermore, given the prevalence and associated personal and societal costs of endometriosis-associated pain (1), attention should be given to determining effective medical strategies for treating neuropathic-like pain in these women. To date, whilst there have been trials in chronic pelvic pain (44, 45), none of the available neuropathic adjunctive analgesics (e.g., amitriptyline, gabapentin, etc.) have been tested in a cohort of exclusively participants with endometriosis-associated pain, despite being relatively cheap and well-tolerated. Any such study should also take into account sensory symptom profiles.

### Limitations of the Study

As participants were recruited from patient support groups, our sample might not necessarily be representative of the patient population. Although our findings may not reflect the picture for women at their first presentation, the long duration of pain and repeated surgical procedures we observed (Table 1) are commonly described for women with endometriosis (4, 41, 46). Therefore, we believe these findings are relevant for the large number of women with persistent/recurrent pain after standard treatment.

Note that a rating of NRS 0 for dyspareunia could either reflect that patients did not experience pain during intercourse or that they have not had intercourse. However, because only 10.2% of responders provided a rating of NRS 0, we believe that this difference does not affect the group-level interpretation of this data.

Given that data were acquired using an online survey, the known limitations of this type of data applies, importantly, that we were unable to independently verify the diagnosis of endometriosis. Although we only included survey respondents whose diagnosis of endometriosis had been confirmed by laparoscopic surgery and who had provided the age at which they received the diagnosis (following surgery), both criteria still relied on self-report. Due to the online format, we were also unable to confirm our findings in a clinical examination. Pain categorisation into nociceptive, mixed and neuropathic based on the painDETECT questionnaire has been shown to agree with clinical examinations in other pain syndromes in the past. However, in-person follow-up studies with clinical examinations are needed to verify our findings and further explore the influence of additional variables (e.g., patient race, stage or location of the disease, endometriosis characteristics, regularity of menstrual cycle, comorbidities such as other pain conditions or uterine/pelvic pathologies) which were not assessed here.

Most importantly, only longitudinal studies will allow us to characterise specific underlying pathological processes and establish causality between these changes and the patients' clinical presentation.

## Conclusion

The data presented here indicate that endometriosis-associated pain includes a neuropathic-like component in a substantial proportion of women. Our findings challenge the current conceptualisation of endometriosis-associated pain as nociceptive and advocates for a new perspective on this type of pain, which is so debilitating to a large number of women.

## Data Availability Statement

The raw data supporting the conclusions of this article will be made available by the authors, without undue reservation.

## Ethics Statement

The studies involving human participants were reviewed and approved by Central University Research Ethics Committee, University of Oxford, R56567/RE002. The patients/participants provided their written informed consent to participate in this study.

## Author Contributions

LC and KV conceived the project. LC collected the data. LC, KV, and KW analysed the data and prepared the manuscript. All authors contributed to the article and approved the submitted version.

## Conflict of Interest

KW has received Consultancy fees from P&G Health, Germany. KV declares that she has received research funding from Bayer AG, Honoraria from Eli Lilly and Honoraria and Consultancy fees from Bayer AG, Grünenthal GmBH and AbbeVie. The remaining author declares that the research was conducted in the absence of any commercial or financial relationships that could be construed as a potential conflict of interest.

## Publisher's Note

All claims expressed in this article are solely those of the authors and do not necessarily represent those of their affiliated organizations, or those of the publisher, the editors and the reviewers. Any product that may be evaluated in this article, or claim that may be made by its manufacturer, is not guaranteed or endorsed by the publisher.
