# Supplementary material for: Is There a Neuropathic-Like Component to Endometriosis-Associated Pain? Results From a Large Cohort Questionnaire Study
Source: Front Pain Res (Lausanne). 2021 Nov 4;2:743812. doi: 10.3389/fpain.2021.743812 (PMC8915551; doi:10.3389/fpain.2021.743812)
Supplement: Supplementary file 1 [file Data_Sheet_1.PDF]

Endometriosis-associated Pain

Page 1

General Information

This study aims to investigate endometriosis-associated pain.

We appreciate your interest in participating in this questionnaire/online survey. Please read through these terms before agreeing to participate by ticking 'yes' box below. You may ask any questions before taking part by contacting the researcher (details below).

We (the University of Oxford) are investigating endometriosis-associated pain.

You will be given some questions / scenarios to read, and then answer questions on. It should take about 10 minutes. No background knowledge is required.

Do I have to take part?

Please note that your participation is voluntary. You may withdraw at any point during the questionnaire for any reason, before submitting your answers, by pressing the 'exit' button / closing the browser.

How will your data be used?

Your answers will be completely anonymous, and we will use all reasonable endeavours to keep them confidential.

Your data will be stored in a password-protected file and may be used in academic publications. Your IP address will not be stored. All questions are optional. Research data will be stored for a minimum of 3 years after publication or public release.

Who will have access to your data?

The University of Oxford is the data controller for the purposes of the Data Protection Act 1998. Your information may be shared with collaborators on this project.

Responsible members of the University of Oxford, funders may be given access to data for monitoring and/or audit of the study to ensure we are complying with guidelines, or as otherwise requires by law.

This questionnaire is for an DPhil project. The principle research is Lydia Coxon, who is attached to the Departement of Women's and Reproductive Health at the University of Oxford. This project is being completed under the supervision of Katy Vincent.

This project has been reviewed by, and received ethics clearance through, the University of Oxford Central University Research Ethics Committee.

What is there is a problem?

If you have a concern about any aspect of this project please speak to the researcher Lydia Coxon (lydia.coxon@st-hughs.ox.ac.uk) or their supervisor Katy Vincent (katy.vincent@obs-gyn.ox.ac.uk), who will do their best to answer your query. The researcher should acknowledge your concern within 10 working days and give you an indication of how they intend to deal with it. If you remain unhappy or wish you make a formal complaint, please contact the relevant Chair of the Research Ethics Committee at the University of Oxford:

Chair, Medical Sciences Inter-Divisional Research Ethics Committee; Email: ethics@medsci.ox.ac.uk; Address: Research Services, University of Oxford, Wellington Square, Oxford OX1 2JD

The Chair will seek to resolve the matter in a reasonably expeditious manner.

Please note that you may only participate in this survey if you are 18 years of age or over.

Please tick this box if you are over the age of 18. ✱ *Required*

- ◌ I certify that I am 18 years of age or over.

If you have read the information above and agree to participate with the understanding that the data you submit will be processed accordingly, please check the relevant box below to get started. ✱ *Required*

- ○ Yes, I agree to take part.

Page 2

How old are you currently (years)?

How old were you when you were diagnosed with endometriosis (years)?

At what age did you start having period pain (years)? Please write N/A if you have not experienced period pain.

Please rate how severe your pelvic pain during your period was at its worst in the last 12 months using a scale from 0 to 10 (where 0 = no pain and 10 = worst imaginable pain).

Please don't select more than 1 answer(s) per row.

	0	1	2	3	4	5	6	7	8	9	10	
no pain	☐	☐	☐	☐	☐	☐	☐	☐	☐	☐	☐	worst imaginable pain

How old were you when you first experienced pain during urination (having a wee) (years)? Please write N/A if you have not experienced pain during urination.

Please rate how severe your pelvic pain during urination (having a wee) was at its worst in the last 12 months using a scale from 0 to 10 (where 0 = no pain and 10 = worst imaginable pain).

Please don't select more than 1 answer(s) per row.

	0	1	2	3	4	5	6	7	8	9	10	
no pain	☐	☐	☐	☐	☐	☐	☐	☐	☐	☐	☐	worst imaginable pain

How old were you when you first experienced pain during bowel movement (having a poo) (years)? Please write N/A if you have not experienced pain during bowel movement.

Please rate how severe your pelvic pain during bowel movement (having a poo) was at its worst in the last 12 months using a scale from 0 to 10 (where 0 = no pain and 10 = worst imaginable pain).

Please don't select more than 1 answer(s) per row.

	0	1	2	3	4	5	6	7	8	9	10	
no pain	☐	☐	☐	☐	☐	☐	☐	☐	☐	☐	☐	worst imaginable pain

Have you ever had pelvic pain during intercourse (sex) or in the 24 hours following vaginal sexual intercourse/penetration?

- Yes
- No

If yes: At what age did this pain start (years)?

[ ]

Please rate how severe your pelvic pain during intercourse (sex) or in the 24 hours following vaginal sexual intercourse/penetration was at its worst using a scale from 0 to 10 (where 0 = no pain and 10 = worst imaginable pain).

Please don't select more than 1 answer(s) per row.

	0	1	2	3	4	5	6	7	8	9	10	
no pain	☐	☐	☐	☐	☐	☐	☐	☐	☐	☐	☐	worst pain imaginable

Have you ever experienced pelvic pain in general? Do not count: pain caused by period pain, intercourse, surgery, pregnancy, childbirth, sports-related or other injury, food poisoning, or stomach flu.

- Yes
- No

If yes: At what age did you start having this pelvic pain (years)?

[ ]

Please rate how severe your pelvic pain in general was at its worst in the last 12 months using a scale from 0 to 10 (where 0 = no pain and 10 = worst imaginable pain).

Please don't select more than 1 answer(s) per row.

	0	1	2	3	4	5	6	7	8	9	10	
no pain	☐	☐	☐	☐	☐	☐	☐	☐	☐	☐	☐	worst imaginable pain

Page 3

How many of each of these operations have you had on your tummy which have involved a cut: (please give a number)

Laparoscopy	
Appendix removal	
Caesarean section	
Burning/laser to endometriosis lesion	
Cutting out of endometriosis lesion	
Other (please specify)	
Other (please specify)	

Page 4

Please select the picture that best describes the course of your pain:

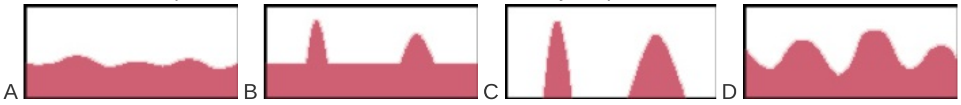

- A Persistent pain with slight fluctuations
- B Persistent pain with pain attacks
- C Pain attacks without pain between them
- D Pain attacks with pain between them

Do you suffer from a burning sensation (e.g. stinging nettles) in the area(s) of your pain?

- never
- hardly noticed
- slightly
- moderately
- strongly
- very strongly

Do you have a tingling or pricking sensation in the area of your pain (like crawling ants or electrical tingling)?

- never
- hardly noticed
- slightly
- moderately
- strongly
- very strongly

Is light touching (clothing, a blanket) in this area painful?

- never
- hardly noticed
- slightly
- moderately
- strongly
- very strongly

Do you have sudden pain attacks in the area of your pain, like electric shocks?

- never
- hardly noticed
- slightly

- moderately
- strongly
- very strongly

Is cold or heat (bath water) in this area occasoinally painful?

- never
- hardly noticed
- slightly
- moderately
- strongly
- very strongly

Do you suffer from a sensation of numbness in this areas?

- never
- hardly noticed
- slightly
- moderately
- strongly
- very strongly

Does slight pressure in this area, e.g. with a finger, trigger pain?

- never
- hardly noticed
- slightly
- moderately
- strongly
- very strongly

Does your pain radiate to other regions of your body?

Page 5

This section contains a series of questions in which you should imagine yourself in certain situations. You should then decide if these situations would be painful for you and if yes, how painful they would be.

Let 0 stand for no pain; 1 is an only just noticeable pain and 10 the most severe pain that you can imagine or consider possible. Please select on the scale the number that is most true for you. Keep in mind that there are no "right" or "wrong" answers; only your personal assessment of the situation counts. Please try as much as possible not to allow your fear or aversion of the imagined situations affect your assessment of painfulness.

Imagine you bump your shin badly on a hard edge, for example, on the edge of a glass coffee table. How painful would that be for you?

Please don't select more than 1 answer(s) per row.

	0	1	2	3	4	5	6	7	8	9	10	
not at all painful	☐	☐	☐	☐	☐	☐	☐	☐	☐	☐	☐	most severe pain imaginable

Imagine you burn your tongue on a very hot drink.

Please don't select more than 1 answer(s) per row.

	0	1	2	3	4	5	6	7	8	9	10	
not at all painful	☐	☐	☐	☐	☐	☐	☐	☐	☐	☐	☐	most severe pain imaginable

Imagine your muscles are slightly sore as the result of physical activity.

Please don't select more than 1 answer(s) per row.

	0	1	2	3	4	5	6	7	8	9	10	
not at all painful	☐	☐	☐	☐	☐	☐	☐	☐	☐	☐	☐	most severe pain imaginable

Imagine you trap your finger in a drawer.

Please don't select more than 1 answer(s) per row.

	0	1	2	3	4	5	6	7	8	9	10	
not at all painful	☐	☐	☐	☐	☐	☐	☐	☐	☐	☐	☐	most severe pain imaginable

Imagine you take a shower with lukewarm water.

Please don't select more than 1 answer(s) per row.

	0	1	2	3	4	5	6	7	8	9	10	

not at all painful												most severe pain imaginable

Imagine you have mild sunburn on your shoulders.

Please don't select more than 1 answer(s) per row.

	0	1	2	3	4	5	6	7	8	9	10	
not at all painful												most severe pain imaginable

Imagine you grazed your knee falling off your bicycle.

Please don't select more than 1 answer(s) per row.

	0	1	2	3	4	5	6	7	8	9	10	
not at all painful												most severe pain imaginable

Imagine you accidentally bite your tongue or cheek badly while eating.

Please don't select more than 1 answer(s) per row.

	0	1	2	3	4	5	6	7	8	9	10	
not at all painful												most severe pain imaginable

Imagine walking across a cool tiled floor with bare feet.

Please don't select more than 1 answer(s) per row.

	0	1	2	3	4	5	6	7	8	9	10	
not at all painful												most severe pain imaginable

Imagine you have a minor cut on your finger and inadvertently get lemon juice in the wound.

Please don't select more than 1 answer(s) per row.

	0	1	2	3	4	5	6	7	8	9	10	
not at all painful												most severe pain imaginable

Imagine you prick your fingertip on the thorn of a rose.

Please don't select more than 1 answer(s) per row.

	0	1	2	3	4	5	6	7	8	9	10	
not at all painful	☐	☐	☐	☐	☐	☐	☐	☐	☐	☐	☐	most severe pain imaginable

Imagine you stick your bare hands in the snow for a couple of minutes or bring your hands in contact with snow for some time, for example, while making snowballs.

Please don't select more than 1 answer(s) per row.

	0	1	2	3	4	5	6	7	8	9	10	
not at all painful	☐	☐	☐	☐	☐	☐	☐	☐	☐	☐	☐	most severe pain imaginable

Imagine you shake hands with someone who has a normal grip.

Please don't select more than 1 answer(s) per row.

	0	1	2	3	4	5	6	7	8	9	10	
not at all painful	☐	☐	☐	☐	☐	☐	☐	☐	☐	☐	☐	most severe pain imaginable

Imagine you shake hands with someone who has a very strong grip.

Please don't select more than 1 answer(s) per row.

	0	1	2	3	4	5	6	7	8	9	10	
not at all painful	☐	☐	☐	☐	☐	☐	☐	☐	☐	☐	☐	most severe pain imaginable

Imagine you pick up a hot pot by inadvertently grabbing its equally hot handles.

Please don't select more than 1 answer(s) per row.

	0	1	2	3	4	5	6	7	8	9	10	
not at all painful	☐	☐	☐	☐	☐	☐	☐	☐	☐	☐	☐	most severe pain imaginable

Imagine you are wearing sandals and someone with heavy boots steps on your foot.

Please don't select more than 1 answer(s) per row.

	0	1	2	3	4	5	6	7	8	9	10	
not at all painful	☐	☐	☐	☐	☐	☐	☐	☐	☐	☐	☐	most severe pain imaginable

Imagine you bump your elbow on the edge of a table ("funny bone").

Please don't select more than 1 answer(s) per row.

	0	1	2	3	4	5	6	7	8	9	10	
not at all painful	☐	☐	☐	☐	☐	☐	☐	☐	☐	☐	☐	most severe pain imaginable

Page 6

Using the following scale, indicate for each item your severity over the past week by checking the appropraite box.

Please don't select more than 1 answer(s) per row.

	No problem	Mild	Moderate	Severe
Fatigue	☐	☐	☐	☐
Trouble thinking or remembering	☐	☐	☐	☐
Waking up tired (unrefreshed)	☐	☐	☐	☐

Have your problems with these symptoms been present for 3 months or more?

During the past 6 months have you had any of the following symptoms:

Please don't select more than 1 answer(s) per row.

	Yes	No
Pain or cramps in the lower abdomen	☐	☐
Depression	☐	☐
Headache	☐	☐

Please check all areas of your body where you have felt persistent or recurrent pain present for the last 3 months or longer (chronic pain) as

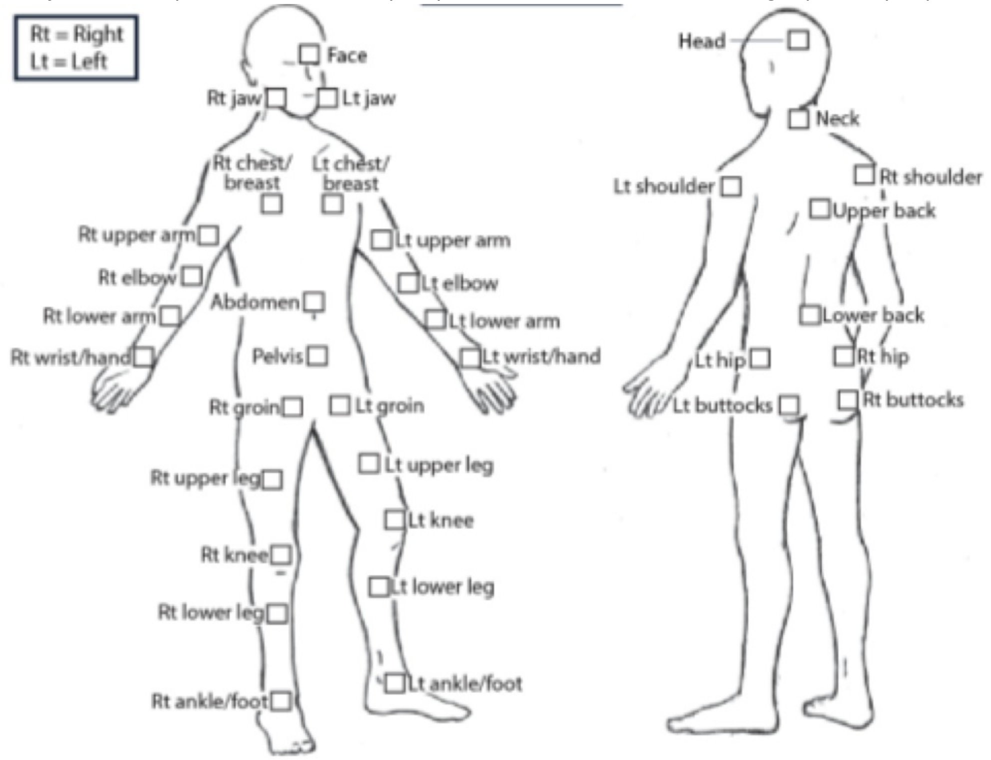

illustrated on the diagram below.

☐ No chronic pain
☐ Face

- ☐ Right jaw
- ☐ Left jaw
- ☐ Right chest/breast
- ☐ Left chest/breast
- ☐ Right upper arm
- ☐ Left upper arm
- ☐ Right elbow
- ☐ Left elbow
- ☐ Right lower arm
- ☐ Left lower arm
- ☐ Right wrist/hand
- ☐ Left wrist/hand
- ☐ Abdomen
- ☐ Pelvis
- ☐ Right groin
- ☐ Left groin
- ☐ Right upper leg
- ☐ Left upper leg
- ☐ Right knee
- ☐ Left knee
- ☐ Right lower leg
- ☐ Left lower leg
- ☐ Right ankle/foot
- ☐ Left ankle/foot
- ☐ Head
- ☐ Neck
- ☐ Left shoulder
- ☐ Right shoulder
- ☐ Upper back
- ☐ Lower back
- ☐ Left hip
- ☐ Right hip
- ☐ Left buttocks
- ☐ Right buttocks

Page 7

Everyone experiences painful situations at some point in their lives. Such experiences may include headaches, tooth pain, joint or muscle pain. People are often exposed to situations that may cause pain such as illness, injury, dental procedures or surgery. We are interested in the types of thoughts and feeling that you have when you are in pain. Listed below are thirteen statements describing different thoughts and feelings that may be associated with pain. Using the scale, please indicate the degree to which you have these thoughts and feelings when you are experiencing pain.

Please don't select more than 1 answer(s) per row.

	Not at all	To a slight degree	To a moderate degree	To a great degree	All the time
I worry all the time about whether the pain will end	☐	☐	☐	☐	☐
I feel I can't go on	☐	☐	☐	☐	☐
It's terrible and I think it's never going to get any better	☐	☐	☐	☐	☐
It's awful and I feel that it overwhelms me	☐	☐	☐	☐	☐
I feel I can't stand it anymore	☐	☐	☐	☐	☐
I become afraid that the pain will get worse	☐	☐	☐	☐	☐
I keep thinking of other painful events	☐	☐	☐	☐	☐
I anxiously want the pain to go away	☐	☐	☐	☐	☐
I can't seem to keep it out of my mind	☐	☐	☐	☐	☐
I keep thinking about how badly I want the pain to stop	☐	☐	☐	☐	☐
There's nothing I can do to reduce the intensity of the pain	☐	☐	☐	☐	☐
I wonder whether something serious may happen	☐	☐	☐	☐	☐

Page 8

The following section consists of 21 groups of statements. Please read each group of statements carefully, and then pick out the one statement in each group that best describes the way you have been feeling during the past two weeks, including today. Please select the statement. If several statements in the group seem to apply equally well, circle the highest number for that group. Be sure that you do not choose more than one statement for any group, including Changes in Sleeping Pattern or Changes in Appetite.

Sadness

- 0 I do not feel sad
- 1 I feel sad much of the time
- 2 I am sad all of the time
- 3 I am so sad or unhappy that I can't stand it

Pessimism

- 0 I am not discouraged about my future
- 1 I feel more discouraged about my future than I used to be
- 2 I do not expect things to work out for me
- 3 I feel my future is hopeless and will only get worse

Past Failure

- 0 I do not feel like a failure
- 1 I have failed more than I should have
- 2 As I look back, I see a lot of failures
- 3 I feel I am a total failure as a person

Loss of Pleasure

- 0 I get as much pleasure as I ever did from the things I enjoy
- 1 I don't enjoy things as much as I used to
- 2 I get very little pleasure from the things I used to enjoy
- 3 I can't get any pleasure from the things I used to enjoy

Guilty Feelings

- 0 I don't feel particularly guilty
- 1 I feel guilty over many things I have done or should have done
- 2 I feel quite guilty most of the time
- 3 I feel guilty all of the time

Punishment Feelings

- 0 I don't feel I am being punished
- 1 I feel I may be punished
- 2 I expect to be punished
- 3 I feel I am being punished

Self-Dislike

- 0 I feel the same about myself as ever
- 1 I have lost confidence in myself
- 2 I am disappointed in myself
- 3 I dislike myself

Self-Criticalness

- 0 I don't criticise or blame myself more than usual
- 1 I am more critical of myself than I used to be
- 2 I criticise myself for all of my faults
- 3 I blame myself for everything bad that happens

Suicidal Thoughts or Wishes

- 0 I don't have any thoughts of killing myself
- 1 I have thoughts of killing myself, but I would not carry them out
- 2 I would like to kill myself
- 3 I would kill myself if I had the chance

Crying

- 0 I don't cry anymore than I used to
- 1 I cry more than I used to
- 2 I cry over every little thing
- 3 I feel like crying, but I can't

Agitation

- 0 I am no more restless or wound up than usual
- 1 I feel more restless or wound up than usual
- 2 I am so restless or agitated the it's hard to stay still
- 3 I am so restless or agitated that I have to keep moving or doing something

Loss of Interest

- 0 I have not lost interest in other people or activities
- 1 I am less interested in other people or things than before
- 2 I have lost most of my interest in other people or things
- 3 It's hard to get interested in anything

Indecisiveness

- 0 I make decisions about as well as ever
- 1 I find it more difficult to make decisions than usual
- 2 I have much greater difficulty in making decisions than I used to
- 3 I have trouble making any decisions

Worthlessness

- 0 I do not feel I am worthless
- 1 I don't consider myself as worthwhile and useful as I used to
- 2 I feel more worthless as compared to other people
- 3 I feel utterly worthless

Loss of Energy

- 0 I have as much energy as ever
- 1 I have less energy than I used to have
- 2 I don't have enough energy to do very much
- 3 I don't have enough energy to do anything

Changes in Sleeping Pattern

- 0 I have not experienced any change in my sleeping pattern
- 1a I sleep somewhat more than usual
- 1b I sleep somewhat less than usual
- 2a I sleep a lot more than usual
- 2b I sleep a lot less than usual
- 3a I sleep most of the day
- 3b I wake up 1-2 hours early and can't get back to sleep

Irritability

- 0 I am no more irritable than usual
- 1 I am more irritable than usual

- 2 I am much more irritable than usual
- 3 I am irritable all the time

18 / 20

Changes in Appetite

- 0 I have not experienced any change in my appetite
- 1a My appetite is somewhat less than usual
- 1b My appetite is somewhat greater than usual
- 2a My appetite is much less than before
- 2b My appetite is much greater than usual
- 3a I have no appetite
- 3b I crave food all the time

Concentration Difficulty

- 0 I can concentrate as well as ever
- 1 I can't concentrate as well as usual
- 2 It's hard to keep my mind on anything for very long
- 3 I find I can't concentrate on anything

Tiredness or Fatigue

- 0 I am no more tired or fatigued than usual
- 1 I get more tired or fatigues more easily than usual
- 2 I am too tired or fatigued to do a lot of the things I used to do
- 3 I am too tired or fatigued to do most of the things I used to do

Loss of Interest in Sex

- 0 I have not noticed any recent change in my interest in sex
- 1 I am less interested in sex than I used to be
- 2 I am much less interested in sex now
- 3 I have lost interest in sex completely

Page 9

Below are a set of statements people have used to describe themselves. Please select the appropriate box to indicate how much each statement applies to how you generally feel.

Please don't select more than 1 answer(s) per row.

	Not at all	Somewhat	Moderately	Very well
I feel pleasant	☐	☐	☐	☐
I feel nervous and restless	☐	☐	☐	☐
I feel satisfied with myself	☐	☐	☐	☐
I wish I could be as happy as others seem to be	☐	☐	☐	☐
I feel like a failure	☐	☐	☐	☐
I feel rested	☐	☐	☐	☐
I am "cool, calm and collected"	☐	☐	☐	☐
I feel that difficulties are piling up so that I cannot overcome them	☐	☐	☐	☐
I worry too much over something that doesn't really matter	☐	☐	☐	☐
I am happy	☐	☐	☐	☐
I have disturbing thoughts	☐	☐	☐	☐
I lack self-confidence	☐	☐	☐	☐
I feel secure	☐	☐	☐	☐
I make decisions easily	☐	☐	☐	☐
I feel inadequate	☐	☐	☐	☐
I am content	☐	☐	☐	☐
Some unimportant thought runs through my head and bothers me	☐	☐	☐	☐
I take disappointments so keenly that I can't put them out of my mind	☐	☐	☐	☐
I am a steady person	☐	☐	☐	☐
I get in a state of tension or turmoil as I think over my recent concerns or interests	☐	☐	☐	☐

Page 10

Thank you very much for completing this survey. We very much appreciate the time you have taken.

Key for selection options

19 - Does your pain radiate to other regions of your body?

Yes
No

38 - Have your problems with these symptoms been present for 3 months or more?

Yes
No
